# Michigan State Board of Health

**Published:** 1879-09

**Authors:** 


### ARTICLE XIII.

### MICHIGAN STATE BOARD OF HEALTH.

The quarterly meeting of the State Board of Health was held in the office of the secretary in the new capitol at Lansing, Tuesday, July 8, 1879. The following members were present: R. C. Kedzie, M.D., of Lansing, President; Homer O. Hitchcock, M.D., of Kalamazoo; Hon. LeRoy Parker, of Flint; Rev. Daniel C. Jacokes, D.D., of Pontiac; Henry F. Lyster, M.D., of Detroit; John H. Kellogg, M.D., of Battle Creek; and Henry B. Baker, M.D., Secretary.

#### PRESIDENT'S ADDRESS.

President Kedzie gave a brief history of the legislation relative to illuminating oils in this State, beginning with the law of 1869. This law provided for county inspection, but was not generally enforced. The legislature of 1873, which passed the law for establishing the State board of health, also passed a law raising the flash test for oil to 150°F. The state board of health began its work with this law in force. In 1875 the legislature reduced the flash test to 140° F., and increased the inspection fees. There were scarcely any casualties under this law, but the illuminating qualities of the oil were not always good. Dr. Kedzie, as a committee of the State board of health, devised the chill test, which was recommended to and adopted by the legislature of 1877, and secured a good and safe illuminating oil. The legislature of 1879 abolished the chill test and reduced the flash test to 120° F. Each time the law has been changed the cost of inspection has been increased, and the last law will entail an annual expense of about $12,000 for inspection above the expenses incurred under the law of 1877.

#### INTERESTED FOREIGNERS.

The president presented a letter from Theodore H. Monk of the meteorological office at Toronto, asking for a set of reports of this board, as they desire to inaugurate a system of health and weather observations similar to that of the Michigan board.

Secretary Baker presented a communication from the secretary of the epidemiological society of London, expressing great interest in the work of the Michigan board, especially that for the registration of disease.

A letter was presented from Mr. Avery of Baltimore, relative to

LEAD POISONING

as set forth by Dr. Kedzie's articles on that subject, and claiming that he had demonstrated that electroplating the tin cans used in preserving fruit, and tin utensils of all kinds, with a thin coating of silver would prevent any poisoning thereby.

CATTLE DISEASES.

A communication was presented from A. J. Murray, veterinary surgeon at Detroit, relative to "cattle diseases in Michigan," and their relation to public health; also, a part of a letter from a member of the national board of health on a similar subject. These communications were referred to the new standing committee on "diseases of domestic animals as relates to public health."

THE SECRETARY'S REPORT.

Secretary Baker presented his report of the work in the office during the last three months. It included the distribution of a large number of the regular reports and other documents, and of the registration report of births, marriages and deaths. These were sent to meteorological observers, regular correspondents, sanitary exchanges, and other persons interested in such subjects in Michigan. Names and addresses of health officers were received from 760 townships, 113 villages, 39 cities. Abstracts of the proceedings of the last meeting were prepared and sent to nine sanitary journals, who desired the same for publication. These journals are exchanges of the board. Meteorological observations were regularly taken in the office of the board, and a condensed statement is each week published in the Lansing *Republican*. Weekly reports from over 60 observers of diseases have been received, examined and filed. Work on the compilation of these reports and of the meteorological reports has been continuously going on. The correspondence of the office is continually increasing, 606 pages of the letter-book being used in copying letters. Quite a number of meteorological instruments have been purchased and sent to observers, and some new stations have been established. A demand for weekly reports of diseases has been made on health officers of cities, as fast as the names have been furnished by the city recorders. The secretary has spent considerable time in supervising vital statistics, particularly those for 1877, and in studying deaths from certain diseases in a series of years. Many persons have visited the office of the board during the past three months, and most of them express surprise at the magnitude of the work carried on by the board. Communications have been received and referred to the chairmen of appropriate committees, as follows: Dr. Kedzie, 14, Dr. Hitchcock 16, Leroy Parker 4, Dr. Jacokes 1, Dr. Lyster 8.

The board has in mind the examination of candidates in sanitary science, and the examination papers on this subject used in the university of London and other foreign colleges have been secured for study in this connection, and Dr. Lyster reported a plan for the examination of physicians in sanitary science.

The standing committees were reorganized, as follows:

*Epidemic diseases, etc.* — Dr. H. O. Hitchcock.

*Sewerage and drainage* — Dr. H. F. Lyster.

*Food, drinks and water supply* — Dr. R. C. Kedzie.

*Ventilation, heating, etc.* — Dr. D. C. Jacokes.

*Climate, etc., in relation to health* — Dr. H. F. Lyster.

*Disposal of decomposing organic matter* — Dr. J. H. Kellogg.

*Poisons, chemicals, accidents, etc.* — Dr. R. C. Kedzie.

*Occupation, etc., in relation to health* — Dr. J. H. Kellogg.

*Relations of schools to health, etc.* — Dr. D. C. Jacokes.

*Sanitary survey* — Dr. Jacokes, Dr. H. B. Baker, and Leroy Parker.

*Death-rate* — Dr. Baker.

*Legislation* — Leroy Parker.

*Finances of the board* — Leroy Parker.

*Mental hygiene* — Dr. Hitchcock.

*Diseases of animals* — Dr. Baker.

Dr. Hitchcock made a report on depot privies, which included letters from the late Dr. Beech, of Coldwater, and J. E. Curtis, superintendent of the Michigan division of the L. S. & M. S. railroad, and made specific recommendations for remedying the nuisances which now prevail. Depot privies should never have a vault, but should be water closets connected with a sewer, or be supplied with dry earth or coal ashes; and it should be made the special duty of a station employe to see that the floors are scrubbed daily, the closets kept clean and in perfect operating order, and the whole closet thoroughly disinfected each day. In places where a sewer is not accessible, the closet in which the dry earth or coal ashes is used should be often cleaned, and the refuse buried. For water closets he recommended "Rhoads' porcelain seated hopper closet," supplied with Meyers' No. 1 waste preventing cistern. This closet is arranged to flush when the door is opened, and is just the thing for public places, as the hopper is non-absorbent, and the shape prevents persons using it from getting on it with their feet. For smaller stations, where a water closet could not be used, he described and recommended an exceedingly simple dry earth closet, but insisted upon the necessity of every day attention to it by an employe of the station.

The committee on sanitary conventions recommended that one be held in Detroit in December or January, and the next at Grand Rapids. Efforts will be made to get as large an exhibition of sanitary appliances together as possible. Manufacturers and dealers in sanitary appliances are requested to forward catalogues, advertisements, etc., and to correspond with the secretary relative to placing their wares on exhibition.

A SAMPLE OF RED FLANNEL, from Dr. Nash, of Lapeer, reported to have caused sores, had been examined by Dr. Kedzie, and found to have been colored with analine which contained arsenic and tin.

REPORTS OF COMMITTEES.

Leroy Parker made a report as to the proper method of bringing suit in cases of nuisances; also, relative to collecting the statistics for the next U. S. census, and relative to authority of the board of health to kill horses afflicted with the glanders.

Dr. Kedzie made a report relative to the proceedings of the sanitary council of the Mississippi valley, held at Memphis, and in conjunction with the national board of health at Atlanta. He gave an extended account of the discussions, notably of that on quarantine. He spoke of a conversation with Dr. Billings of the national board of health, in which the following statements were made by Dr. Billings: The quarantine to be established by the national board of health must be uniform for the whole country. It is therefore necessary to be very guarded in the action of the national board, lest requirements which are essential for New Orleans and Mobile may destroy the commerce of New York and Boston. It is proposed to make such sanitary regulations as may and should be enforced in all places, and only such *national restrictions* by quarantine as will not disturb commerce seriously, and, for any stringent quarantine in points especially threatened, to secure action by State and local quarantine.

A resolution was adopted, favoring the organization of sanitary associations auxilliary to local boards of health.

The usual number of bills were audited, and ordinary business transacted. The next meeting of the board will be on October 14, 1879, at nine a. m.

---

EPITAPH ON DR. JOHN LOW, Founder of the Faculty of Physicians and Surgeons. Obiit 1612.

"Who cured Many while He lieved,
So gracious, He no Man Grieved.
Yea, when as Physick's Force oft fayled,
His Pleasant Purpose then prevayled.
For of His God He Gott the Grace
To Live in Mirth and Die in Peace.
Heaven Hes His Soul, His Corps this Stone,
Sygh Passinger, and soe Begone."

DR. GEORGE W. ELKINS, of Chester, Illinois, has recently been appointed surgeon to Illinois Southern Penitentiary.

